# On the Action of Chloroform

**Published:** 1852-08

**Authors:** 


### MATERIA MEDICA AND THERAPEUTICS.

*On the Action of Chloroform.* By M. HERVEZ DE CHEGOIN.—M. Hervez de Chégoin thinks it probable that chloroform may in some cases exert a deleterious influence on the organs, short of death; and he relates a case of amputation of the thigh, in which anæsthesia was produced, and in which both the mucous membrane and the divided surface of the muscles presented a blue appearance. The bone became denuded of periosteum, the suppuration became unhealthy, and the patient died. He would not, however, give up the use of chloroform; but would employ it until the patients manifested a desire to remove themselves from its influence, pushing it away with the hand, at the same time that they have a noise in the ears, and they feel a pinch but obtusely. He has operated on several occasions under these circumstances, the patients not being aware of the operation.—*Ibid.*

